# Syndecan-3 as a Novel Biomarker in Alzheimer’s Disease

**DOI:** 10.3390/ijms23063407

**Published:** 2022-03-21

**Authors:** Anett Hudák, Annamária Letoha, Csaba Vizler, Tamás Letoha

**Affiliations:** 1 Pharmacoidea Ltd., H-6726 Szeged, Hungary; anett.hudak@pharmacoidea.eu; 2 Albert Szent-Györgyi Clinical Center, Department of Medicine, Faculty of Medicine, University of Szeged, H-6720 Szeged, Hungary; letohadr@gmail.com; 3 Biological Research Centre, Institute of Biochemistry, H-6726 Szeged, Hungary; vizler.csaba@brc.hu

**Keywords:** Alzheimer’s disease, biomarkers, syndecan-3, brain, blood–brain barrier, blood, monocytes

## Abstract

Early diagnosis of Alzheimer’s disease (AD) is of paramount importance in preserving the patient’s mental and physical health in a fairly manageable condition for a longer period. Reliable AD detection requires novel biomarkers indicating central nervous system (CNS) degeneration in the periphery. Members of the syndecan family of transmembrane proteoglycans are emerging new targets in inflammatory and neurodegenerative disorders. Reviewing the growing scientific evidence on the involvement of syndecans in the pathomechanism of AD, we analyzed the expression of the neuronal syndecan, syndecan-3 (SDC3), in experimental models of neurodegeneration. Initial in vitro studies showed that prolonged treatment of tumor necrosis factor-alpha (TNF-α) increases SDC3 expression in model neuronal and brain microvascular endothelial cell lines. In vivo studies revealed elevated concentrations of TNF-α in the blood and brain of APPSWE-Tau transgenic mice, along with increased SDC3 concentration in the brain and the liver. Primary brain endothelial cells and peripheral blood monocytes isolated from APPSWE-Tau mice exhibited increased SDC3 expression than wild-type controls. SDC3 expression of blood-derived monocytes showed a positive correlation with amyloid plaque load in the brain, demonstrating that SDC3 on monocytes is a good indicator of amyloid pathology in the brain. Given the well-established role of blood tests, the SDC3 expression of monocytes could serve as a novel biomarker for early AD detection.

## 1. Introduction

Alzheimer’s disease (AD), a disorder characterized by the abnormal accumulation of misfolded protein aggregates and subsequent neuronal death, is the leading cause of dementia worldwide [1,2,3,4,5,6]. Due to the lack of efficient therapeutics, AD is yet untreatable [7,8]. Therefore, AD patients have to face progressive mental and physical deterioration [9]. As currently available symptomatic treatments do not decelerate or prevent the progression of the disease, early diagnosis is one of our most promising tools to tackle AD [10,11,12]. The development of early diagnostics against AD is a key to detecting the disease in the stage of reasonably mild central nervous system (CNS) degeneration [13,14]. The discovery of predictive biomarkers is thus essential for developing accurate AD diagnostics [15,16]. The discovery of such biomarkers requires a profound understanding of early pathophysiological changes leading to neurodegeneration [17,18,19]. Early pathophysiological changes could predict the onset of the disease when the patient is still in a manageable mental and physical condition, thus enabling the application of treatments that could halt the progression of AD [20,21,22].

Previously we explored the contribution of syndecans (SDCs), a family of transmembrane heparan sulfate proteoglycans (HSPGs), to the seeding and spreading of amyloid-beta (Aβ) and tau aggregates [23,24,25]. According to our studies, overexpression of SDCs, especially the neuronal SDC3, creates favorable conditions for the cellular accumulation and subsequent aggregation of misfolded proteins. Seeding and spreading of pathological protein aggregates, on the other hand, has paramount importance in inducing neurodegeneration [26,27]. The involvement of SDCs in the pathogenesis of AD has also been confirmed in several human studies; the colocalization of SDCs with amyloid plaques has already been observed, along with the correlation between AD pathology and SDC gene expression pattern [28,29,30,31,32,33]. Moreover, the increased expression of the neuronal SDC3 and the more ubiquitous SDC4 in postmortem human AD brains and APP/PS1 mice have already been reported [34].

The four members (SDC1-4) of the SDC family have also been implied in the pathogenesis and regulation of inflammation [35]. Due to their versatile glycosaminoglycan (GAG) heparan sulfate (HS) and chondroitin sulfate (CS) chains, SDCs interact with a myriad of inflammatory molecules (including, but not limited to cytokines and growth factors), along with facilitating the recruitment and extravasation of leukocytes to the inflammation site [36]. The role of SDC3 on endothelial cells is emerging in inflammatory responses [37]. SDC3 expressed on inflamed vascular endothelia binds leukocytes and chemokines during the progression of rheumatoid arthritis [38,39]. In the brain, endothelial cells’ SDCs also modulate the transendothelial migration of monocytes, thus contributing to the formation of neuroinflammatory lesions [40]. On the other hand, CNS-infiltrating monocytes play essential roles in AD by eliminating circulating Aβ microaggregates and brain Aβ plaques [41,42,43,44]. 

Inflammation is a central mechanism in AD, driving disease pathology and progression [45,46]. Cytokines and other inflammatory mediators are shown in the brains of AD patients [47]. Tumor necrosis factor-alpha (TNF-α) is also elevated in AD brains and cerebrospinal fluid from AD patients [48,49,50]. Recent reports also show significantly higher levels of proinflammatory cytokines, including TNF-α, in peripheral blood samples of AD patients [51,52]. Induction of monocyte SDC3 was found to be increased when monocytes were cultured with TNF-α in vitro [53]. Furthermore, SDC3 expression was also massively induced in inflammatory monocytes in vivo [53].

SDC3 (N-SDC or neuronal SDC) is a 442 amino acid long transmembrane protein expressed mainly by neurons [54]. SDC3 has four conserved glycosaminoglycan-binding sites at the N-terminus and unique threonine-rich and mucin-like regions close to the membrane. The HS chains of SDC3 serve as binding sites for several chemokines and growth factors, hence playing a role in inflammation and regulating memory and body weight/metabolism [37,55,56,57,58,59,60,61].

Reviewing the growing scientific evidence on the involvement of SDC3 in inflammation and neurodegeneration, we decided to analyze SDC3 in experimental models of inflammation and AD. Our results show that the AD-induced overexpression of SDC3, not just in the brain but also in the periphery, could serve as the base for developing future diagnostics against AD.

## 2. Results

### 2.1. TNF-α Increased SDC3 Expression of SH-SY5Y and hCMEC/D3 Cell Lines

**Figure 1:**
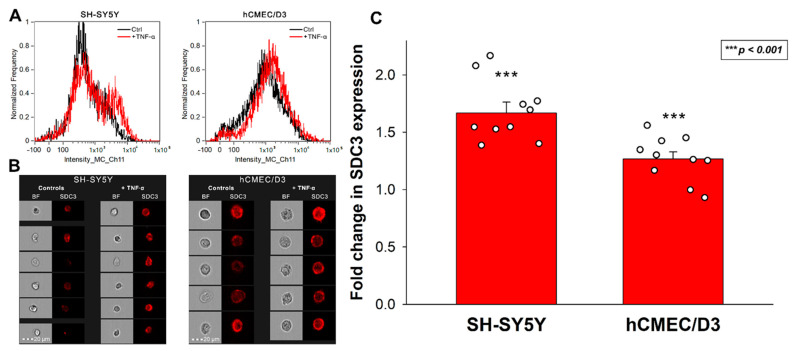
SDC3 expression of TNF-α-treated SH-SY5Y and hCMEC/D3 cells. Cells were incubated with or without (i.e., controls) 5 ng/mL TNF-α for 7 days. After incubation, the cells were treated with APC-labeled SDC3 antibody, and SDC3 was measured with imaging flow cytometry. (**A**) Representative flow cytometry histograms showing the SDC3 expression of SH-SY5Y and hCMEC/D3 cells. (**B**) Brightfield (BF) and fluorescent cellular images of SH-SY5Y and hCMEC/D3 cells treated with APC-labeled SDC3 antibody. Scale bar = 20 μm. (**C**) Detected fluorescence intensities were normalized control cells untreated with TNF-α. The bars represent the mean + SEM of ten independent experiments. Statistical significance vs. controls was assessed with ANOVA. *** *p* < 0.001.

As a critical mediator of the inflammatory response, TNF-α is one of the most well-defined proinflammatory cytokines in AD pathogenesis [50,62]. Evidence suggests that Aβ plaques induce microglial activation and TNF-α release, triggering CNS inflammation that plays a deleterious role in neuronal death [62,63,64]. Several studies also demonstrated that elevated blood TNF-α in AD patients is strongly associated with the rate of cognitive decline [49,65,66,67]. Considering the scientific evidence on the importance of TNF-α in AD, we decided to analyze the effect of TNF-α on SDC3 expression of model cell lines representing two essential classes of cells in AD. SH-SY5Y represented neuronal-like cells widely used in neurobiology, while hCMEC/D3 is a gold standard cell line to study blood–brain barrier-derived endothelial cells [68,69]. Both cell lines have endogenous SDC3 expression (Supplementary Figure S1), and incubating them with TNF-α for seven days increased their SDC3 expression. SDC3 overexpression due to TNF-α was significantly increased in the two cell lines. The effect was most profound in SH-SY5Y cells and slightly less, still significant in HCMEC/D3 cells (Figure 1A–C).

### 2.2. SDC3 Expression in Transgenic Mice Model of AD

**Figure 2:**
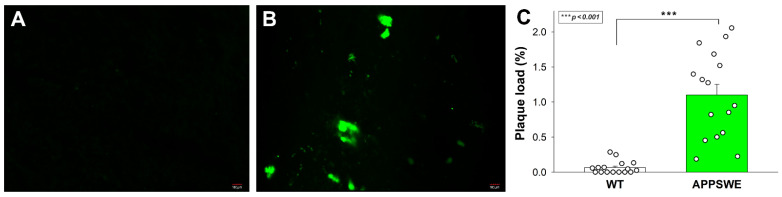
APPSWE-Tau mice exhibits significantly increased amyloid plaque load. (**A**,**B**) Representative brain slices WT (**A**) and APPSWE-Tau mice (**B**) stained with Aβ1-42 antibody. Scale bar = 100 μm. (**C**) The amyloid plaque load was quantified on Aβ1-42 antibody–stained frontal brain slices from 12-month-old APPSWE-Tau and WT mice. Each group contained 8 animals; the plaque load was measured in two slices of each animal. Data are expressed as mean + SEM. *** *p* < 0.001.

APPSWE-Tau is a double mutant transgenic mice model that develops neurofibrillary tangles and progressive motor disturbance and expresses mutant beta-amyloid precursor protein (APP), thus modulating the APP-Aβ environment [70]. APPSWE-Tau mice thus successfully exhibit both hallmark pathologies in AD, and the interaction between Aβ and tau pathologies in APPSWE-Tau mice excellently mimics human AD [71]. In our studies, 12-month-old APPSWE-Tau mice, compared with wild type (WT) C57BL/6 mice, exhibited significantly increased amyloid plaque load (Figure 2A–C).

**Figure 3:**
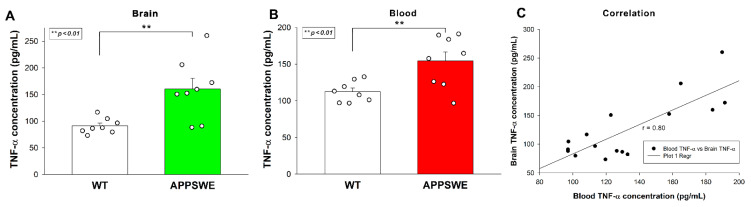
APPSWE-Tau mice exhibits increased TNF-α concentrations in the brain and the blood. (**A**,**B**) TNF-α concentrations of brain extracts (**A**) and whole blood (**B**) of APPSWE-Tau mice, along with representative WT controls, were measured with a mouse SDC3 ELISA kit. Each group contained 8 animals. The bars represent the mean + SEM. Statistical significance vs. WT was assessed with ANOVA. ** *p* < 0.01. (**C**) Linear regression between the TNF-α content of blood and brain.

Increased Aβ plaque load was also associated with elevated TNF-α concentrations in APPSWE-Tau mice’s brain samples, as shown by ELISA measurements (Figure 3A). TNF-α blood concentrations of APPSWE-Tau were also significantly higher than WT controls (Figure 3B). TNF-α concentration in the blood and the brain exhibited a strong correlation with a covariance of 0.80 (Figure 3C).

**Figure 4:**
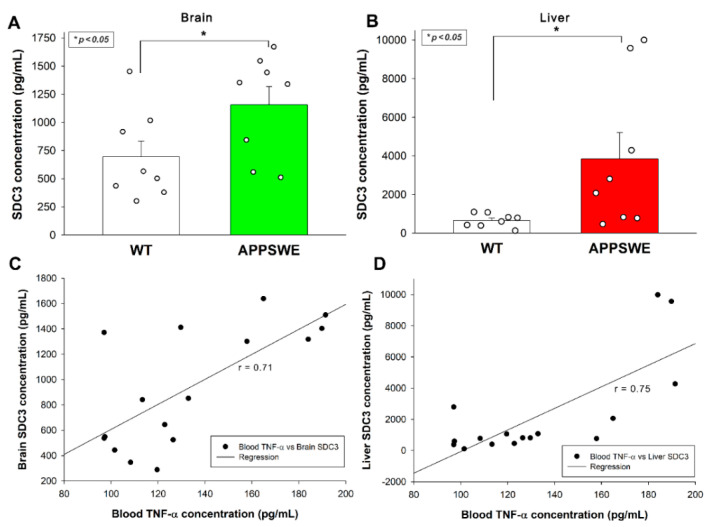
APPSWE-Tau mice exhibits increased SDC3 concentrations in the brain and the liver. (**A**,**B**) SDC3 concentrations of the brain (**A**) and liver (**C**) extracts of APPSWE-Tau mice and representative WT controls were measured with ELISA. Each group contained 8 animals. The bars represent the mean + SEM. Statistical significance vs. WT was assessed with ANOVA. * *p* < 0.05. (**C**,**D**) Linear regression between the TNF-α and SDC3 concentrations in the brain (**C**) and liver (**D**).

ELISA measurements also revealed increased SDC3 concentrations in the brain (Figure 4A) and the periphery, namely the liver (Figure 4B). As shown in Figure 4C,D, increased SDC3 concentrations in the brain and the liver correlated well with blood TNF-α concentrations, suggesting a cause and effect relationship between TNF-α and SDC3 expression, as shown in previous in vitro studies.

**Figure 5:**
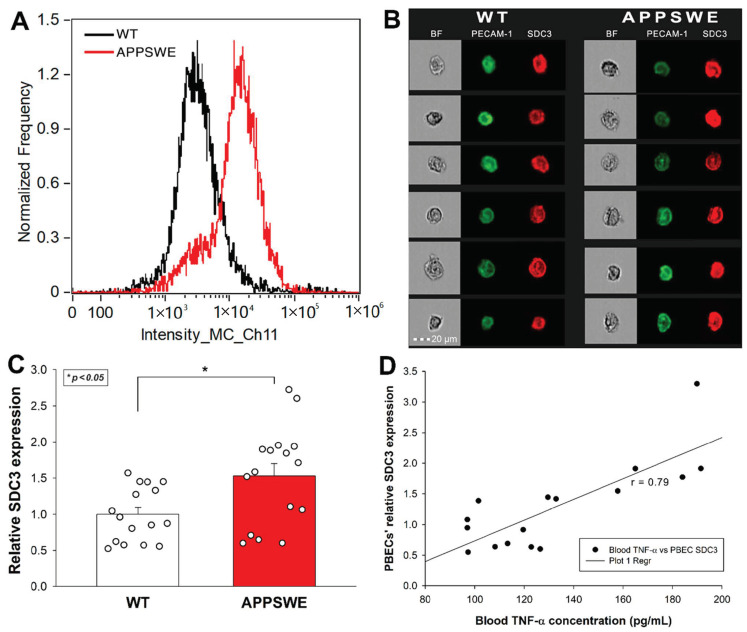
PBECs of APPSWE-Tau mice exhibits increased SDC3 expression. Isolated PBECs were treated with PECAM-1 and SDC3 antibodies, and SDC3 expression of PECAM-1 positive cells was analyzed with imaging flow cytometry. (**A**) Representative histogram showing the SDC3 expression of PBECs isolated from APPSWE-Tau and WT mice. (**B**) BF and fluorescent cellular images of PECAM-1 and SDC3 antibody-treated PBECs, isolated from APPSWE-Tau and WT mice. Each group contained eight animals. SDC3 expression of each sample was measured twice. (**C**) Detected fluorescence intensities were normalized to WT. The bars represent the mean + SEM. Statistical significance vs. WT was assessed with ANOVA. * *p* < 0.05. (**D**) Linear regression between in PBECs’ relative SDC3 expression and blood TNF-α concentrations.

Considering the emerging role of endothelial cells’ SDC3 in the inflammatory response, along with our initial in vitro data on TNF-α-induced SDC3 expression in human blood–brain barrier (BBB) endothelial cells, we also analyzed the SDC3 expression of primary BBB endothelial cells isolated from mice. Primary brain endothelial cells (PBECs) were isolated with the method of Assmann et al., and SDC3 expression was analyzed with imaging flow cytometry using fluorescent SDC3 antibodies [72]. A fluorescently labeled PECAM-1 (platelet endothelial cell adhesion molecule-1) antibody was used as an endothelial marker to identify PBECs during the flow cytometry analyses [73]. As Figure 5A–C shows that the SDC3 expression of PBECs isolated from APPSWE-Tau was significantly increased compared with those isolated from WT mice.

**Figure 6:**
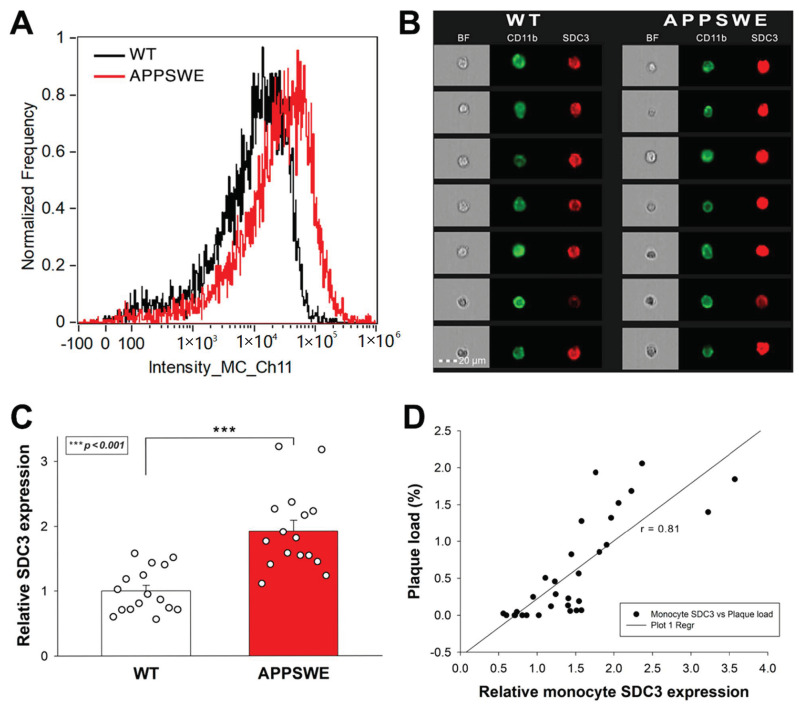
Monocytes isolated from APPSWE-Tau mice exhibits increased SDC3 expression. Isolated monocytes were treated with CD11b and SDC3 antibodies, and SDC3 expression of CD11b positive cells was analyzed with imaging flow cytometry. (**A**) Representative histogram showing the SDC3 expression of monocytes isolated from APPSWE-Tau and WT mice. (**B**) BF and fluorescent cellular images of CD11b and SDC3 antibody-treated monocytes, isolated from APPSWE-Tau and WT mice. Each group contained eight animals. SDC3 expression of each sample was measured twice. (**C**) Detected fluorescence intensities were normalized to WT. The bars represent the mean + SEM. Statistical significance vs. WT was assessed with ANOVA. *** *p* < 0.001. (**D**) Linear regression between the SDC3 expression of monocytes and Aβ plaque loads.

The roles of SDC3 on leukocytes are emerging in inflammatory responses [37]. TNF-α induces the SDC3 expression of cultured monocytes. Furthermore, SDC3 is also massively induced on inflammatory monocytes in vivo in sections of inflamed synovia from the joints of patients with rheumatoid arthritis [74]. Considering the role of monocytes in the progression of AD, we also analyzed SDC3 expression of monocytes isolated from the blood of APPSWE-Tau and WT mice. Thus blood-derived monocytes were isolated with EasySep™ Mouse Monocyte Isolation Kit, stained with CD11b monocyte marker, and the SDC3 expression of CD11b positive cells was measured with imaging flow cytometry [75]. Monocytes isolated from APPSWE-Tau mice exhibited increased SDC3 expression than those from WT mice (Figure 6A–C). SDC3 expression of blood-derived monocytes showed a positive correlation (r = 0.81) with Aβ plaque deposition in the brain, showing that SDC3 on monocytes is a good indicator of amyloid pathology in the brain (Figure 6C).

## 3. Discussion

AD is the leading cause of senile dementia [76]. AD patients are usually diagnosed in a stage of cognitive deficits with underlying CNS dementia [9,77]. Due to the irreversible nature of neurodegeneration, patients diagnosed with symptomatic AD progress into gradual mental and physical deterioration. As curative AD therapeutics are lacking, early AD diagnosis has paramount importance in preserving a patient’s level of function for a more extended period [11,12,13]. The emergence of AD-specific biomarkers improved diagnostic specificity [15,78]. Newly discovered biomarkers allow AD diagnosis before the onset of dementia, namely in the stage of mild cognitive impairment (MCI), which has variously been termed prodromal AD in the presence of amyloid biomarkers [15,79]. The medical practice still requires more AD biomarkers to validate their usefulness in the clinics [15,80,81]. Given the ongoing worldwide AD epidemics and the inequality of available high-tech laboratory instrumentation, one of the most critical requirements for AD biomarkers is easy detection in low-cost diagnostic settings [80,81]. The widespread use of blood testing makes blood-based biomarkers ideal for AD diagnosis [80]. Blood-based biomarkers can improve detection and low-cost diagnosis of AD by the ease of testing. Blood-based tests enable the detection of a wide range of exploratory and candidate pathophysiological biomarkers, reflecting the full spectrum of AD-driving molecular mechanisms beyond the conventional amyloid- and tau-based tests [80]. Blood-based biomarkers could facilitate a more profound understanding of AD molecular pathophysiology and accelerate the development of disease-modifying therapies [80,81].

SDCs are emerging molecular targets in AD [24,25,33,34,82]. Postmortem human AD brains show increased SDC3 and four expressions, while increased SDC expression correlates with regional vulnerabilities of AD brains [33,34]. Previously we have shown that SDC overexpression, especially the neuronal SDC3, triggers the seeding and cellular spreading of misfolded protein aggregates, including Aβ [24,25]. Meanwhile, other studies demonstrated the proinflammatory role of SDC3 in inflammation [35,36,37,74].

Considering the evidence on the contribution of SDC3 to inflammation and neurodegeneration, we decided to analyze the changes of SDC3 expression due to proinflammatory and neurodegenerative stimuli. In vitro, TNF-α significantly increased SDC3 expression in the neuronal-like SH-SY5Y and BBB-derived hCMEC/D3 cells. In the transgenic mice model of AD, APPSWE-Tau, increased Aβ plaque load was associated with elevated blood and brain TNF-α concentrations (those correlated with each other, too), a clear indicator of the inflammatory aspects of the ongoing neurodegeneration. In correlation with the increased blood TNF-α, SDC3 concentrations also increased in the CNS (i.e., brain) and the periphery (i.e., liver), suggesting that neurodegeneration-related inflammation also extends to the periphery. PBECs isolated from APPSWE-Tau mice also exhibited higher SDC3 expression than WT mice, highlighting the molecular changes occurring in the BBB during the progress of AD pathology. Monocytes isolated from peripheral blood of APPSWE-Tau mice also showed increased SDC3 expression that correlated with CNS plaque load. As SDC3 monocytes isolated from the systemic circulation reflect amyloid pathology, SDC3 expression of monocytes could serve as a blood-based biomarker diagnosing AD.

In summary, our data confirm the expression changes of SDC3, a proteoglycan with an established role in protein aggregation and inflammation, in preclinical models of AD. The detected increase in SDC3 expression in both the brain and the periphery correlates with the increased TNF-α concentrations, an established indicator of inflammation. Inflammation associated with neurodegeneration also affects BBB, as reflected by the increased SDC3 expression of BBB-derived primary endothelial cells. The selective expression of endothelial SDC3 was already explored in the chronically inflamed synovium, where SDC3 plays a part in arthritis pathophysiology by binding cytokines and modulating the migration and retention of leukocytes [38,39]. In the BBB, SDC3 modulates the transendothelial migration of monocytes [40]. An increase in endothelial SDC3 could thus demonstrate a novel link between BBB vascular changes and neuroinflammation during AD pathogenesis, thus facilitating peripheral monocytes migrating into the brain to phagocytose Aβ plaques [41,42,43,44]. A recent study revealed distinct phenotypic and functional changes in monocyte and macrophage populations as AD progress [83]. As detected in our studies, the increased SDC3 expression of blood monocytes confirms monocyte activation due to systemic inflammation associated with AD. The correlation of SDC3 expression changes of peripheral monocytes with Aβ pathology highlights the relevance of monocytes’ SDC3 as a predictive biomarker of AD progression. Further clinical studies should confirm our findings obtained in preclinical models. However, considering the involvement of SDC3 in inflammatory conditions in general, other established AD biomarkers should also supplement the utilization of SDC3 as a peripheral biomarker of AD pathology.

## 4. Materials and Methods

### 4.1. Flow Cytometry Analysis of Cellular SDC3 Expression

SDC3 expression of SH-SY5Y and hCMEC/D3 cells incubated with or without 5 ng/mL of recombinant TNF-α (cat. no. 210-TA-100/CF, RnD Systems, Minneapolis, MN, USA) for 7 days was measured with an AMNIS FlowSight imaging flow cytometer (Luminex Corporation, Austin, TX, USA) by using APC-labeled anti-human SDC3 antibody (polyclonal goat IgG, cat. no. FAB3539A, RnD Systems) as described previously [24,25]. Goat IgG APC-conjugated antibody (RnD Systems, cat. no. IC108A) was used as an isotype control.

SDC3 expression of primary mouse brain endothelial cells and monocytes was analyzed with imaging flow cytometry using mouse SDC3 antibody and specific monocyte or endothelial markers (CD11b or PECAM-1). After isolation, the isolated primary cells were incubated with primary SDC3 antibody (cat. no. sc-398194, Santa Cruz Biotechnology, Inc., Dallas, TX, USA) and fluorescently labeled secondary antibody (Alexa Fluor 633-labeled goat anti-mouse IgM, cat. no. A-21046, Invitrogen, Waltham, MA, USA). Respective cellular markers (PBECs: mouse PECAM-1 Alexa Fluor 488-conjugated Antibody, cat. no. FAB6874G, RnD Systems and monocytes: Alexa Fluor 488-labeled CD11b Monoclonal Antibody, Invitrogen, cat. no. 53-0112-82) were used to identify PBECs and monocytes. A minimum of 5000 events per sample was analyzed. Appropriate gating was utilized to exclude cellular debris and aggregates. Fluorescence analysis was carried out with the Amnis IDEAS analysis software. 

### 4.2. Animal Experiments

For in vivo experimental studies, APPSWE-Tau (Taconic Biosciences, Inc., Germantown, NY, USA) and healthy C57BL/6 mice (WT) with a minimum age of 12 months were used. The number of animals in each group (i.e., WT and APPSWE-Tau) was 8–8, with an equal number of males (4–4 individuals) and females (4–4 individuals) in both groups. Blood of mice anesthetized with 2,2,2-Tribromoethanol (cat. no. T48402, Merck KGaA, Darmstadt, Germany) was collected with cardiac puncture. After transcardial perfusion with ice-cold PBS (2 mL/min; cat. no. BE17-516F, Lonza, Basel, Switzerland), the brain was removed, dissected frontally, and frozen in dry ice for further ELISA and microscopic examination.

### 4.3. Immunohistochemistry

For immunohistochemistry, mouse brain samples (n = 8 mice per group) were fixed for 18 h in 4% paraformaldehyde (cat. no. P6148, Sigma-Aldrich), then dehydrated in an ethanol series, cleared with xylene (cat. no. 00699464, Avantor Inc., Radnor, PA, USA), and embedded in paraffin (cat. no. 26154.291, Avantor Inc.). Ten μm thick sections were finally cut with a microtome (Leica Biosystems Inc., Buffalo Grove, IL, USA), and sections were collected on SuperFrost Plus^®^ slides (Thermo Fisher Scientific Inc., Waltham, MA, USA). Antigen detection was carried out with heat-induced antigen recovery. Slides were first immersed in citrate buffer heated to 95–100 degrees for 10 min, then cooled to room temperature for about 20 min. Next, the slides were placed in blocking solution (5% goat or donkey serum diluted in 0.1% PBST) at room temperature for 30 min, and then the blocking solution was removed without rinsing. The slides were then incubated with 100 μL of primary antibody (Aβ specific antibody, MOAB-2, cat. no. NBP2-13075, NOVUS Biologicals, Littleton, CO, USA) diluted in blocking solution (1% BSA or goat serum in 0.1% PBST) at room temperature for 1 h or at 4 °C overnight. Slides were then rinsed with PBST 3x for 10 min each at room temperature, followed by staining with 100 μL of Alexa Fluor 488-labeled secondary antibody (cat. no. A-21141, Thermo Fisher Scientific) diluted in blocking solution for 1h at room temperature. The slides were then rinsed 3 timeswith PBS at room temperature for 10 min and, using mounting media, were covered with cover plates.

### 4.4. Plaque Load Assessment

Morphometry for Aβ load determination was performed using ImageJ image processing and analysis software by interactive measurement of plaque areas in the total area of interest. The plaque load was calculated as the percentage of the area of interest covered by amyloid plaques stained with the Aβ specific antibody [84,85]. Plaque load of two samples from each animal were calculated.

### 4.5. Measuring TNF-α Tissue Concentrations

Brain samples were homogenized in lysis buffer (cat. no. 79216, QIAGEN, Düsseldorf, Germany) in 1% NP-40/PBS in cOmplete, Mini, EDTA-free protease inhibitor cocktail (cat. no. 11836170001, Roche, Basel, Switzerland), and tissue lysates were analyzed with mouse TNF-alpha Quantikine ELISA Kit (cat. no. MTA00B, RnD Systems, Minneapolis, MN, USA) according to the manufacturer’s instructions. The TNF-α concentration of whole blood isolated from mice was also measured with the same ELISA kit.

### 4.6. Measuring SDC3 Tissue Concentration

Brain and liver samples were homogenized in lysis buffer (QIAGEN) in 1% NP-40/PBS in Complete Mini EDTA-free protease inhibitor cocktail (Roche). Tissue lysates were analyzed with mouse SDC3 ELISA Kit PicoKine^®^ (cat. no. EK1556, BOSTER Biological Technology, Pleasanton, CA, USA) according to the manufacturer’s instructions.

### 4.7. Isolation of Mouse Monocytes and Brain Endothelial Cells

Monocytes were isolated from the collected blood samples with the EasySep™ Mouse Monocyte Isolation Kit (cat. no. 19861, Stemcell Technologies Inc, Vancouver, BC, Canada) according to the manufacturer’s instructions. Mouse brain endothelial cells were isolated using the method of Assmann et al. [72]. SDC3 expression of isolated monocytes and primary brain endothelial cells (PBECs) was analyzed with imaging flow cytometry using mouse SDC3 antibody and specific monocyte or endothelial markers (CD11b or PECAM-1). After isolation, the isolated primary cells were incubated with primary SDC3 antibody (cat. no. sc-398194, Santa Cruz Biotechnology, Inc., Dallas, TX, USA) and fluorescently labeled secondary antibody (Alexa Fluor 633-labeled goat anti-mouse IgM, cat. no. A-21046, Invitrogen, Waltham, MA, USA). Respective cellular markers (PBECs: mouse PECAM-1 Alexa Fluor 488-conjugated Antibody, cat. no. FAB6874G, RnD Systems and monocytes: Alexa Fluor 488-labeled CD11b Monoclonal Antibody, Invitrogen, cat. no. 53-0112-82) were used to identify PBECs and monocytes.

### 4.8. Statistical Analysis

Results are expressed as means + standard error of the mean (SEM). Differences between experimental groups were evaluated using a one-way analysis of variance (ANOVA). Values of *p* < 0.05 were accepted as significant. Pearson’s correlation coefficient was used to measure the strength of a linear association between two variables.

## Data Availability

Data are contained within the article or Supplementary Materials.

## Supplementary Materials

The following are available online at www.mdpi.com/article/10.3390/ijms23063407/s1.
