# Supplementary material for: Syndecan-3 as a Novel Biomarker in Alzheimer’s Disease
Source: Int J Mol Sci. 2022 Mar 21;23(6):3407. doi: 10.3390/ijms23063407 (PMC8955174; doi:10.3390/ijms23063407)
Supplement: Supplementary file 1 [file ijms-23-03407-s001.zip › ijms-1635067-supplementary-done.pdf]

International Journal of
Molecular Sciences

MDPI

Supplementary Materials: Syndecan-3 as a novel biomarker in Alzheimer's disease

Anett Hudák [1], Annamária Letoha [2], Csaba Vizler [3], László Szilák [1], and Tamás Letoha [1,]

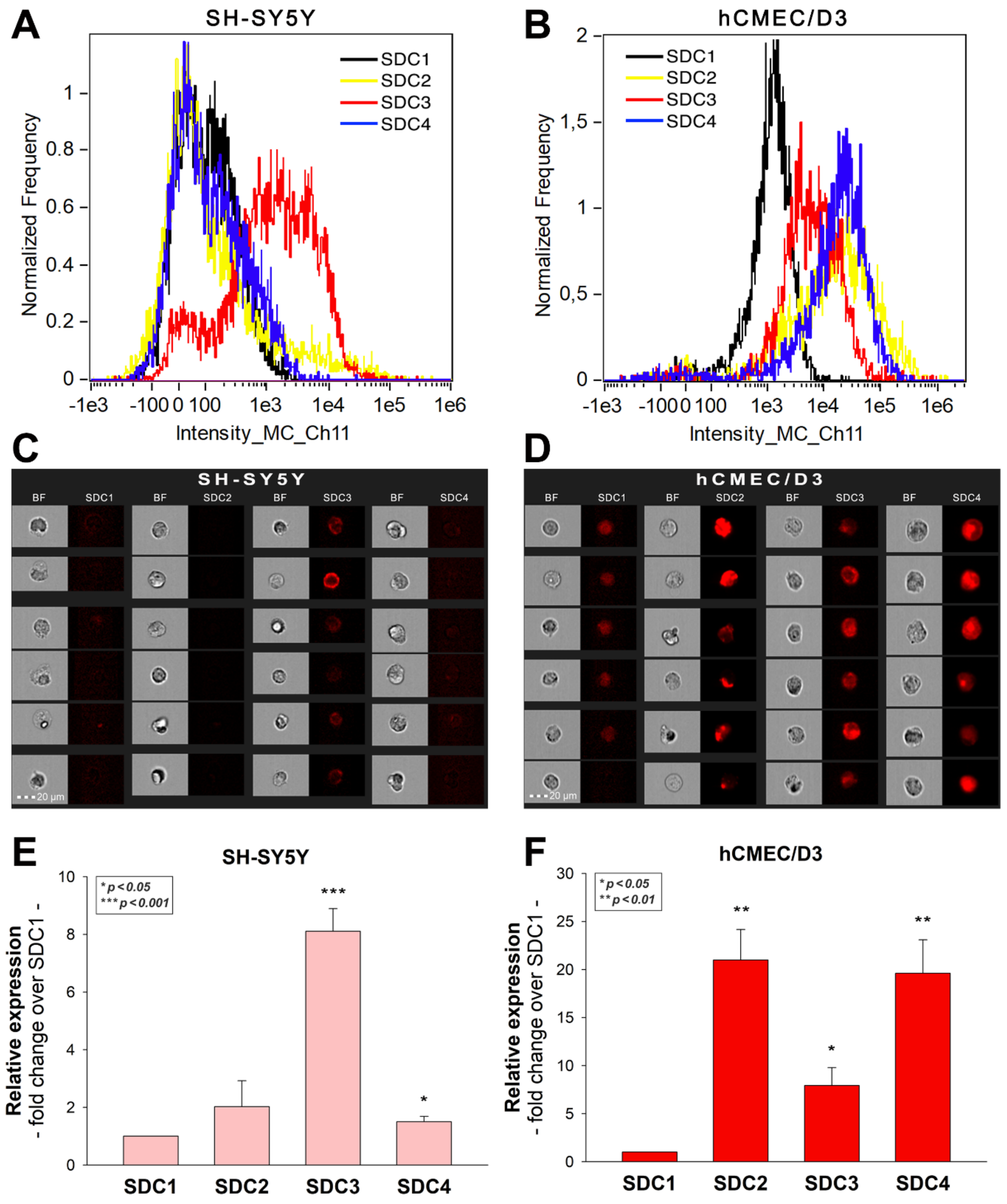

Figure S1. SDC expression profile of SH-SY5Y and hCMEC/D3 cells. SDC1-4 expression of SH-SY5Y and hCMEC/D3 cells was analyzed with imaging flow cytometry using APC-labeled anti-SDC antibodies specific for each SDC isoform. **(A,B)** Representative flow cytometry histograms showing

the SDC1-4 expression of SH-SY5Y and hCMEC/D3 cells. (**C,D)** Brightfield (BF) and fluorescent cellular images of SH-SY5Y and hCMEC/D3 treated with the respective SDC antibodies. Scale bar = 20 μm. (**E,F)** Detected SDC expression levels were normalized to that of SDC1 as standard. The bars represent the mean + SEM of three independent experiments. Statistical significance vs. SDC1 expression levels (standards) was assessed with analysis of variance (ANOVA). *$p<0.05$; **$p<0.01$; ***$p<0.001$.
